# Endoscopic resection combined with gel immersion and curved laryngoscope for superficial hypopharyngeal cancer

**DOI:** 10.1055/a-2099-4084

**Published:** 2023-06-22

**Authors:** Takahiro Inoue, Chikatoshi Katada, Takahiro Shimizu, Mitsuhiro Nikaido, Hirokazu Higuchi, Yo Kishimoto, Manabu Muto

**Affiliations:** 1 Department of Gastroenterology and Hepatology, Graduate School of Medicine, Kyoto University, Kyoto, Japan; 2 Department of Therapeutic Oncology, Graduate School of Medicine, Kyoto University, Kyoto, Japan; 3 Department of Medical Equipment, Kyoto University Hospital, Kyoto, Japan; 4 Department of Otolaryngology-Head and Neck Surgery, Graduate School of Medicine, Kyoto University, Kyoto, Japan

Transoral surgery using gastrointestinal endoscopy for superficial lesions near the pharyngoesophageal junction is an effective treatment 1 2 . Gel immersion endoscopic mucosal resection has been reported to be a promising treatment for superficial lesions of the digestive tract 3 4 5 .

A 59-year-old man who underwent esophagogastroduodenoscopy because of dysphagia exhibited a migrating, protruding lesion at the posterior wall of the hypopharynx near the pharyngoesophageal junction. Endoscopic examination failed to show the entire lesion due to natural constriction by the sphincter and the gag reflex ( Fig. 1 ). The lesion was pathologically diagnosed as squamous cell carcinoma. Because no obvious metastasis was identified, the patient was treated by transoral surgery under general anesthesia.

**Fig. 1:**
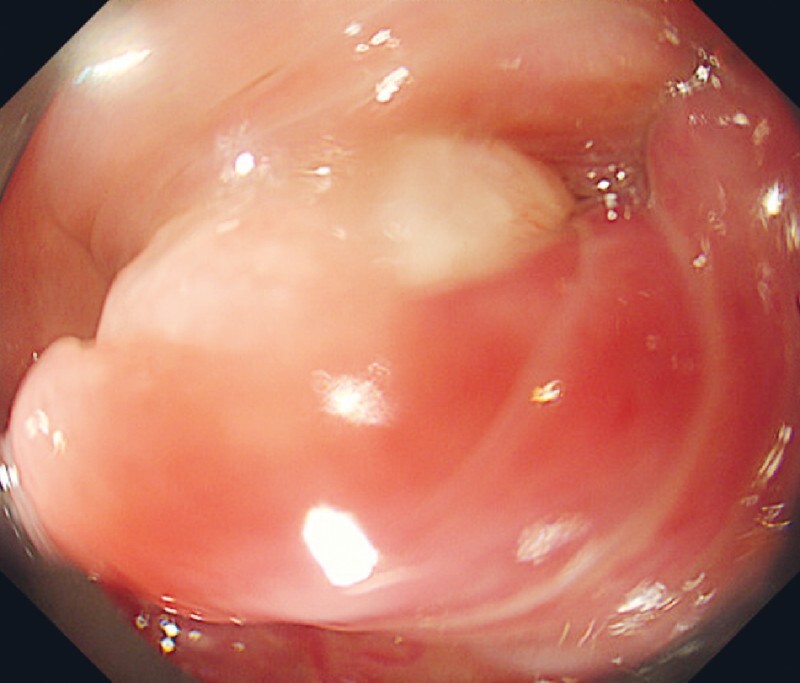
Endoscopic image showing a migrating, protruding lesion at the pharyngoesophageal junction. Natural constriction by the sphincter and the gag reflex presented a challenge in visualizing the entire lesion.

Wide hypopharyngeal exposure using a curved laryngoscope revealed that an 18 mm hypopharyngeal tumor with a stalk shifted to the esophagus. The lesion was pulled into the hypopharynx using grasping forceps ( Fig. 2 ). Magnifying endoscopy with narrow-band imaging showed no superficial extension beyond the base of the stalk. Clear viscous gel (VISCOCLEAR; Otsuka Pharmaceuticals Factory, Tokushima, Japan) was injected into the hypopharyngeal lumen to obtain a clear endoscopic view and maintain the expanded lumen. Lugol chromoendoscopy with gel immersion allowed us to determine the optimal surgical margins. Under gel immersion, sufficient buoyancy was obtained to float the lesion, and the lesion movement was reduced ( Fig. 3 ). Using a bipolar snare (Dragonare 20 mm; Xemex Co., Ltd., Tokyo, Japan) with electrocautery, en bloc resection was achieved within 5 minutes, without adverse events. The histopathological diagnosis confirmed squamous cell carcinoma invading the subepithelial layer, with negative margins and no lymphovascular invasion. No additional treatment was given because there were no risk factors for metastasis ( Fig. 4 , Video 1 ).

**Fig. 2:**
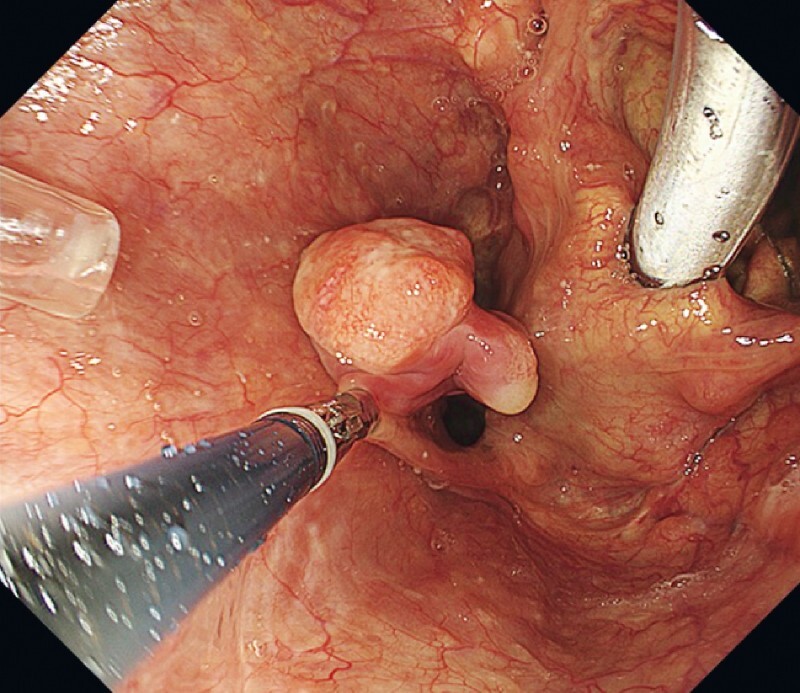
Wide hypopharyngeal exposure using a curved laryngoscope was employed under general anesthesia, and an 18 mm hypopharyngeal tumor with a stalk was confirmed using grasping forceps.

**Fig. 3:**
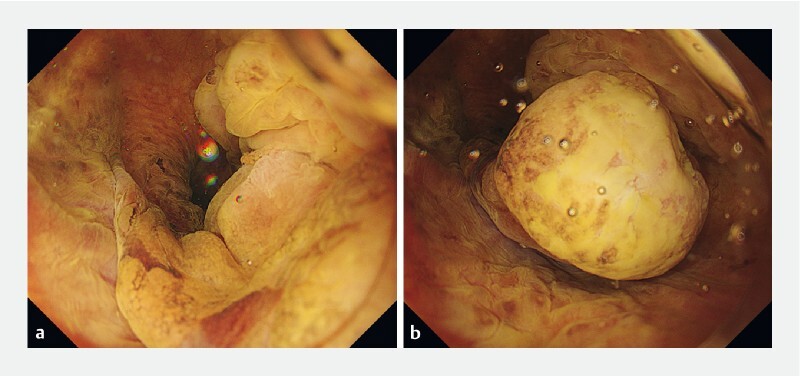
Gel immersion endoscopy provided a favorable endoscopic field of view and sufficient buoyancy to float the lesion, facilitating snare resection. **a** Gel immersion Lugol chromoendoscopy showing the stalk of the lesion. **b** Gel immersion Lugol chromoendoscopy showing the head of the lesion.

**Fig. 4:**
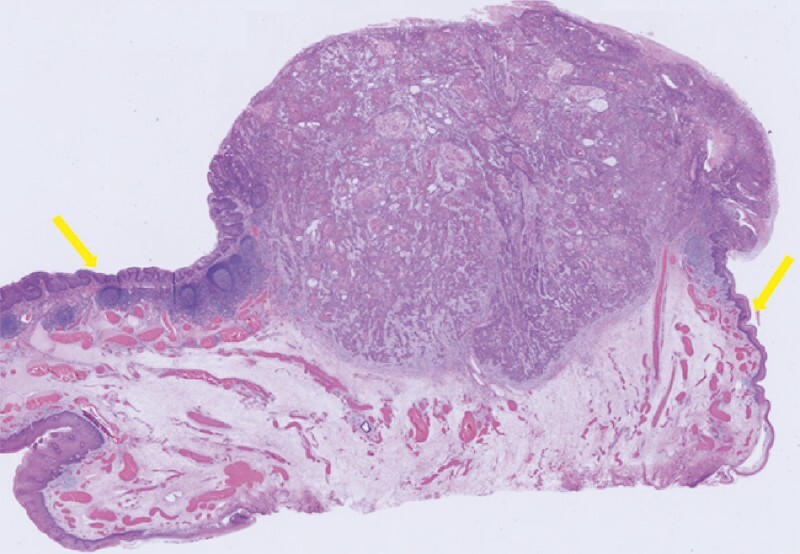
En bloc resection was successfully achieved. The histopathological diagnosis was squamous cell carcinoma invading the subepithelial layer. The horizontal (arrows) and vertical margins were negative, and lymphovascular invasion was not detected.

**Video 1** Endoscopic resection combined with gel immersion and curved laryngoscope provided a favorable endoscopic view and sufficient buoyancy for the lesion with a stalk at the pharyngoesophageal junction, leading to successful resection. Source for graphical illustration: Yasuaki Furue (Department of Gastroenterology, Kitasato University School of Medicine, Sagamihara, Japan).


Endoscopic resection combined with gel immersion and curved laryngoscope may be an effective treatment strategy in locations where the lumen is narrow, such as the pharyngoesophageal junction.

Endoscopy_UCTN_Code_CCL_1AB_2AB

## Notes

Endoscopy E-Videos

https://eref.thieme.de/e-videos
*E-Videos* is an open access online section of the journal *Endoscopy* , reporting on interesting cases and new techniques in gastroenterological endoscopy. All papers include a high-quality video and are published with a Creative Commons CC-BY license. Endoscopy E-Videos qualify for HINARI discounts and waivers and eligibility is automatically checked during the submission process. We grant 100% waivers to articles whose corresponding authors are based in Group A countries and 50% waivers to those who are based in Group B countries as classified by Research4Life (see: https://www.research4life.org/access/eligibility/ ). This section has its own submission website at https://mc.manuscriptcentral.com/e-videos
